# 

**Published:** 1867-01

**Authors:** 


Annual Meeting of the State Medical Society.—The Sixtieth Annual Meeting of the Medical Society of the State of New York, will be held in the city of Albany, February 5th, 6th and 7th, 1867. A full and interesting meeting is confidently expected.

Wm. H. Bailey,
Secretary.

### Books and Pamphlets Received.

The Renewal of Life—Lectures, chiefly Clinical. By Thomas King Chambers, M. D., Honorary Physician to H. R. H. the Prince of Wales. Second American from the fourth London edition. Philadelphia: Lindsay & Blakiston.
For sale by Theodore Butler, 159 Main street. Price $5.

The Functions and Disorders of the Reproductive Organs in Childhood, Youth, Adult Age and Advanced Life, considered in their Physiological, Social and Moral Relations. By William Acton, M. R. C. S. Second American from the fourth London edition. Philadelphia: Lindsay & Blakiston.
For sale by Theodore Butler, 159 Main street. Price $3.

An Index of Diseases and their Treatment. By Thomas Hawkes Tanner, M. D., F. L. S., Member of the Royal College of Physicians, etc. Philadelphia: Lindsay & Blakiston.
For sale by Theodore Butler, 159 Main street. Price $3.

Transactions of the American Medical Association, vol. 17, 1866. Notice of its contents and merits will appear in our next issue.

Case of Luxation of Femur into Ischiatic Notch, of nine months' standing, reduced by manipulation. By Lewis A. Sayre, M. D., Surgeon of Bellevue Hospital, etc., etc.

Treatment of Fracture of the Lower Jaw by Interdental Splints. By Thomas Brian Gunning, New York.

On the Inhalation of Atomized Fluids. By H. Beigel, M. D., L. R. C. P.

Seventeenth Annual Report of the Trustees of the Wisconsin Institution for the Education of the Blind, October, 1866.

Annual Announcement of Lectures in the Atlanta Medical College, for the session of 1867, with a Catalogue of Matriculates in 1865–6.

Commencement Exercises in the Buffalo Medical College.

The commencement exercises of the Medical College will be held at St. James' Hall, on Tuesday evening, February 26th. The address to the graduates will be given by Prof. James P. White. Members of the medical profession are cordially invited to be present. Particulars of the exercises will appear in our next journal.

